# Experiences of Forgone Care During the COVID-19 Pandemic and Older Adults’ Mental Health: Variations by Race and Ethnicity

**DOI:** 10.1007/s40615-025-02304-0

**Published:** 2025-02-11

**Authors:** Jen-Hao Chen, Ming Wen

**Affiliations:** 1 Department of Sociology and Department of Psychology, National Chengchi University, Taipei, Taiwan; 2 Department of Sociology and Research Hub of Population Studies, University of Hong Kong, Hong Kong, China

**Keywords:** Healthcare utilization, Psychological distress, Racial disparities, Sleep

## Abstract

The COVID-19 pandemic led to substantial forgone care. However, relatively few studies have investigated the mental health consequences of forgone care experiences on older adults and how these associations vary by race and ethnicity during this period. This study analyzed US older adults aged 55 or older from the Health, Ethnicity, and Pandemic Survey (*N* = 883; average age = 65), a nationally representative survey conducted in late 2020. Weighted OLS regression was used to examine the association between measures of forgone care and mental health outcomes. Results showed that experiences of forgone care were prevalent and similar across racial and ethnic groups, with some variations across types of missed care. Furthermore, forgone care and missing various types of care during the COVID-19 pandemic were associated with higher psychological distress and poorer sleep quality, with some variations by race and ethnicity. Black older adults who had forgone care and missed more types of care showed even poorer sleep than their White counterparts. Additionally, missing chronic care, mental care, and preventative care, but not other types of care, was associated with greater psychological distress and poorer sleep for all racial and ethnic groups. However, Black older adults who missed chronic care had even poorer sleep than Whites. Findings emphasize the need to support all older adults’ healthcare access of various types during the pandemic and the importance of focusing on specific racial and ethnic groups such as Black older adults who were more affected by forgone care.

**Supplementary Information:**

The online version contains supplementary material available at 10.1007/s40615-025-02304-0.

## Background and Objectives

The COVID−19 pandemic has highlighted the issue of forgone care, defined as delaying or not receiving needed medical services, as the public health crisis significantly altered the context in which individuals seek and receive healthcare. At the macro level, during the initial phase of the pandemic, the US healthcare system experienced major disruptions, with reports of many hospitals operating at less than full capacity because hospitals purposefully curtailed elective surgeries and other noncritical medical services [1, 2]. At the individual level, the fear of contracting diseases led many people to avoid seeking in−person medical care. This is made worse by economic downturns and policy limitations, as noted by Taylor et al. [3] and Ziedan, Simon, and Wing [4]. Additionally, there are social structural factors that influence healthcare utilization even during normal periods.

Older adults, who are more likely to have chronic health conditions and require regular medical care, faced heightened risks of forgone care as they navigated these disruptions. Studies suggest that forgone care among older adults was prevalent, with more than half of US adults aged 50 or older experiencing any forgone care during the early phase of the pandemic [5]. However, studies from the United States and European countries suggest that older adults were less likely to forgo care during the COVID-19 pandemic than young and middle-aged adults [5–7]. Furthermore, studies of adult populations suggest that disadvantaged groups, such as racial minorities, sexual minorities, and economically disadvantaged populations, were more likely to report forgone care during the COVID-19 pandemic [5, 8]. Additionally, geographic inequities, such as living in rural areas with fewer healthcare resources, also played a role in preventing individuals from accessing necessary medical services [9]. Individuals with chronic conditions or mental health issues were found to have higher rates of forgone care [6], which is particularly concerning as these populations are at a greater risk for severe health outcomes if their conditions are not properly managed. However, studies that examine minority older adults’ experiences of forgone care, and which types of care were missed or delayed, especially among racial minorities, remain relatively scarce.

Additionally, while previous studies have established a connection between forgone care and adverse health outcomes, less is known about the potential impact on psychological well-being and sleep quality, both of which are critical indicators of overall health. For example, individuals who choose to forgo necessary care may experience the exacerbation of untreated conditions, higher risk of complications and mortality, as well as increased healthcare costs in the long run [10–12]. However, the consequences for psychological well-being during the pandemic have received relatively little attention [13]. Previous studies have suggested that missed or delayed care can lead to adverse psychological consequences, such as sleep disturbances, for several reasons [14].

First, the chronic pain and discomfort from untreated medical conditions can significantly impact individuals’ psychological states and sleep quality [15, 16]. Chronic pain often leads to increased anxiety and depression, creating a cycle of poor mental health and sleep disturbances. The constant physical discomfort prevents restful sleep, which exacerbates psychological distress, further deteriorating sleep quality. Second, delayed or missed care can result in late diagnosis of illnesses. Patients experiencing symptoms without a clear diagnosis endure substantial stress due to the uncertainty and fear of the unknown [17]. This anxiety about undiagnosed symptoms can lead to heightened psychological distress, which in turn disrupts sleep patterns. The anticipation of potentially severe health issues and the prolonged suffering without a diagnosis can keep individuals awake at night, worrying about their health [18]. Finally, timely access to medical care is crucial for effective disease management [19], which can alleviate psychological distress and improve sleep. Regular medical interventions and treatments help patients manage their conditions more effectively, reducing worries and concerns. When patients feel they have control over their health and are receiving adequate care, their anxiety levels decrease, leading to better mental health and improved sleep quality [20].

Lastly, while there has been prior research on the determinants and consequences of forgone care, very few studies have delved into how these experiences differ across different racial/ethnic groups [9, 20]. The relationship between race/ethnicity and forgone care remains a relatively under-explored topic, particularly with respect to its impact on psychological well-being and sleep quality during the COVID-19 pandemic. Racial and ethnic minorities, including Asian, Black, and Hispanic populations, often face systemic barriers to healthcare access, which can exacerbate the consequences of forgone care. These groups may experience higher levels of psychological distress and poorer sleep quality due to less access to culturally competent care. Moreover, the stigma associated with the pandemic has disproportionately affected minority communities such as Asian communities [21, 22]. The compounded stress from both the pandemic and systemic inequities can further deteriorate mental health and sleep quality in these minoritized populations. Understanding these disparities will help in creating more equitable healthcare policies and practices that can mitigate the psychological and sleep-related consequences of forgone care in times of the public health crisis.

The primary objective of this study is to address the gap in current research by (1) investigating the relationship between a range of forgone care experiences and the psychological well-being of older adults in the United States during the early stages of the COVID-19 pandemic, and (2) exploring how these experiences differ across race and ethnicity. By analyzing these groups separately, we aim to uncover unique patterns and disparities that are often obscured in broader analyses. Our study is one of the first to disaggregate the experiences of forgone care by race/ethnicity among older adults, providing nuanced insights into how these experiences influence psychological well-being and sleep quality. The findings of this study will inform healthcare providers and practitioners about the specific needs of different racial and ethnic groups, enabling the development of tailored strategies to address health disparities.

## Research Design and Methods

### The Health, Ethnicity, and Pandemic Survey

The Health, Ethnicity and Pandemic (HEAP) study collected data from a nationally representative sample of US adults aged 18 and over during October 2020, focusing on minority populations’ experiences, mental health, and healthcare utilization amidst the COVID-19 pandemic. Utilizing a multi-stage, stratified sampling design from the NORC AmeriSpeak Panel, the study oversampled non-Hispanic Blacks, non-Hispanic Asians, and Hispanics to ensure adequate representation. Most respondents completed the survey online, while those without internet access participated via telephone. The survey was available in both English and Spanish for respondents. A total of 2709 respondents were included in the study. This study was conducted following the ethical principles outlined in the Declaration of Helsinki. Approval was obtained from the NORC Institutional Review Board. Written informed consent was obtained from all participants prior to their inclusion in the study. Detailed design and sampling methods are documented in prior publications [9, 23, 24]. For the purposes of this study, we focused on a subset of older adults aged 55 and above, resulting in a sample size of 910. We excluded 27 older adults whose race and ethnicity were classified as others, leading to the final sample size of 883.

### Experiences of Forgone Care

The HEAP study collected data that enabled us to evaluate the prevalence and nature of forgone healthcare. Each respondent was asked if they had delayed or forgone various types of care due to the COVID-19 pandemic. These included (1) preventive care, such as physical examinations, cancer screenings, and flu vaccinations; (2) mental health care or counseling; (3) prescription medicines; (4) dental care; (5) eyeglasses; (6) specialist care; (7) follow-up care; (8) other medical care; (9) or no care delayed. Firstly, we generated a binary measure to indicate whether care had been forgone, which was marked when one or more of the care options were checked and “no care delayed” was not checked. Secondly, we created a summary measure to assess the cumulative impact of missed care across various types. For instance, a score of 2 indicated that the respondent had missed two types of care. Finally, we created a series of binary measures to examine the consequences of missing each type of healthcare, including chronic care, mental care, preventative care, and others.

### Measures of Psychological Well-Being

The dependent variables were two continuous scales measuring respondents’ psychological distress and sleep quality. Psychological distress was assessed using the K6 scale, which includes six items evaluating the frequency of feelings such as nervousness, hopelessness, restlessness, depression, worthlessness, and the perception that everything was an effort over the past 30 days. Responses ranged from 0 (none of the time) to 4 (all of the time), and a composite score, ranging from 0 to 24, was calculated by summing the item scores. Sleep quality was measured using a single-item question asking respondents to rate their sleep quality over the past week on a scale from 0 (terrible) to 10 (excellent). Both scales were treated as continuous variables in the subsequent regression analysis.

### Statistical Analysis

First, descriptive statistics were calculated to describe the sample characteristics and the prevalence of forgone care experiences. Next, weighted Ordinary Least Squares (OLS) regression models were employed to analyze the association between experiences of forgone care and measures of psychological well-being. Our first model, Model 1, adjusted for potential confounders including age, gender, race/ethnicity, educational attainment, marital status, employment status, health insurance coverage status (i.e., no insurance, Medicaid, Medicare, private insurance), political affiliation (i.e., Democrat, lean Democrat, independent or none, lean Republican, Republican), self-rated health, and adherence to personal protective behaviors during the pandemic. Model 2 included interaction terms measures of forgone care and race/ethnicity. All analyses were conducted using survey weights to account for the complex sampling design and to ensure the generalizability of the findings to the US population.

## Results

Table 1 presents weighted descriptive statistics of experiences of forgone care among different racial and ethnic groups. The table reveals that approximately two-thirds of older adults had missed or delayed healthcare due to COVID-19 and the proportion of individuals who reported forgone care is relatively similar across racial and ethnic groups. The accumulation of missed care across various types of care shows slight differences. Hispanics exhibit the highest accumulation, followed by Blacks, Whites, and Asians. When examining the types of care missed or delayed, chronic care emerges as the most frequently missed type across all groups, with Hispanics reporting the highest percentage of missed chronic care. Hispanics also reported the highest percentage of missed mental care. Nevertheless, Blacks and Hispanics had lower rates of missed preventative care compared to Whites and Asians. Table 1 shows that forgone care was prevalent for older adults during the early phase of the pandemic and the data also suggests some racial and ethnic disparities in the experiences of forgone care, especially by type of care missed.

**Table 1:** Weighted descriptive statistics of experiences of forgone care (*N* = 883, weighted)

	Total sample	White	Asian	Black	Hispanic
Forgone care	59.66%	57.55%	57.84%	65.58%	64.45%
Accumulation of missed care across various types	1.29	1.20	1.28	1.44	1.65
Type of care missed or delayed					
Chronic care	44.65%	39.84%	44.45%	49.09%	61.56%
Mental care	2.68%	2.47%	2.10%	1.53%	6.32%
Preventive care	18.64%	21.10%	18.14%	12.90%	13.01%
Other types of care	34.00%	36.58%	35.31%	36.47%	19.11%
Age	65.92 (13.43)	66.45 (9.48)	65.84 (12.59)	64.76 (8.73)	64.52 (7.89)
Sample size	883	243	313	208	119

Mean and standard deviation (in parentheses) were reported for continuous variables, and proportions were reported for categorical variables

Table 2 presents the results from weighted regression analyses examining the relationship between experiences of forgone care during the COVID-19 pandemic and psychological well-being among older adults. Panel A shows results for psychological distress, and Panel B shows results for sleep quality. In Model 1 of Panel A, older adults who experienced forgone care during the pandemic reported significantly higher psychological distress (Coeff. = 1.57, *p* < 0.001) after controlling for a wide range of covariates. In Model 2, the inclusion of interaction terms reveals some variations of the associations between forgone care and psychological distress by race and ethnicity. Interaction terms for Asians and Blacks were not statistically significant, suggesting comparable levels of psychological distress related to forgone care in Asians, Blacks, and Whites. In Panel B, experiencing forgone care was also significantly associated with poorer sleep quality (Coeff. = 0.74, *p* < 0.001 in Model 1), accounting for all covariates. The interaction terms for Asians and Hispanics were not significant. However, the interaction term for Blacks (relative to Whites) was significant (Coeff. = 0.85, *p* < 0.05), indicating that Black older adults experienced a greater decline in sleep quality due to forgone care compared to their White counterparts.

**Table 2:** Results from weighted regression linking experiences of forgone care to mental health outcomes (*N* = 883; weighted)

	Model 1 Coefficient (SE)	Model 2 Coefficient (SE)
Panel A: psychological distress		
Forgone care	1.49*** (0.30)	1.43*** (0.36)
Asian (ref = White) × forgone care		0.89 (1.26)
Black × forgone care		0.95 (0.95)
Hispanic × forgone care		− 1.86^+^ (0.97)
Panel B: poor sleep quality		
Forgone care	0.70*** (0.13)	0.66*** (0.15)
Asian (ref = White) × forgone care		− 0.74 (0.53)
Black × forgone care		0.89* (0.41)
Hispanic × forgone care		− 0.44 (0.42)

Model 1 was the base model. Model 2 included interaction terms between experience of forgone care and measures of psychological well-being. Coefficients for covariates were omitted for brevity

^*^p < 0.05; **p < 0.01; ***p < 0.001; ^+^*p* < 0.1

Moving to Table 3, it shows results from weighted regression analyses examining the relationship between the accumulation of missed care types across various types during the COVID-19 pandemic and psychological well-being among older adults. Similarly, Panel A focuses on psychological distress, and Panel B addresses poor sleep quality. Results from Panel A, Model 1 indicate that the accumulation of missed care across various types positively correlated with greater psychological distress (Coeff. = 0.59, *p* < 0.001). Most of the interaction terms in Model 2 were not significant. Results from Panel B, Model 1 show similar findings: the accumulation of missed care types was also significantly associated with poorer sleep quality (Coeff. = 0.20, *p* < 0.001). In Model 2, the interaction term indicates that the association between the accumulation of missed care types and poorer sleep quality was more pronounced for Black older adults compared to their White counterparts (Coeff. = 0.39, *p* < 0.01). Other interaction terms, however, were not statistically significant.

**Table 3:** Results from weighted regression linking accumulation of missed care across various types to mental health outcomes (*N* = 883; weighted)

	Model 1 Coefficient (SE)	Model 2 Coefficient (SE)
Panel A: psychological distress		
Accumulation of missed care across various types	0.58*** (0.11)	0.57*** (0.13)
Asian (ref = White) × accumulation		0.21 (0.41)
Black × accumulation		0.30 (0.30)
Hispanic × accumulation		− 0.52^+^ (0.29)
Panel B: poor sleep quality		
Accumulation of missed care across various types	0.19*** (0.04)	0.16* (0.05)
Asian (ref = White) × accumulation		− 0.12 (0.17)
Black × accumulation		0.41** (0.13)
Hispanic × accumulation		− 0.04 (0.12)

Model 1 was the base model. Model 2 included interaction terms between accumulation of missed care across various types and measures of psychological well-being. Coefficients for covariates were omitted for brevity

^*^p < 0.05; **p < 0.01; ***p < 0.001; ^+^*p* < 0.1

Finally, Table 4 examines the role of specific types of forgone care on psychological well-being among older adults across racial and ethnic groups. Several patterns stand out. First, in Model 1, forgone chronic care was associated with higher psychological distress (Coeff. = 1.51, *p* < 0.001 in Model 1) and poorer sleep quality (Coeff. = 0.76, *p* < 0.001). Similar findings were found for forgone mental care and forgone preventative care. However, forgone other types of care did not show a significant association with psychological distress (*b* = 0.61, *p* > 0.1 in Model 1) but it did correlate with poorer sleep quality (Coeff. = 0.43, *p* < 0.05). Second, in Model 2, results suggest that Hispanics might experience less psychological distress due to forgone preventive care compared to Whites (Coeff. =  − 6.16, *p* < 0.001). Additionally, Black older adults experienced a greater decline in sleep quality due to forgone chronic care compared to Whites (Coeff. = 1.39, *p* < 0.01). Supplementary figures provide graphic representations of the associations presented in Tables 2, 3, and 4 between measures of forgone care and mental health outcomes by race and ethnicity.

**Table 4:** Results from weighted regression linking different types of missed care to mental health outcomes (*N* = 883; weighted)

	Model 1 Coefficient (SE)	Model 2 Coefficient (SE)
Panel A: psychological distress		
Forgone chronic care (ref = no foregone care)	1.48*** (0.36)	1.18* (0.46)
Forgone mental care	6.03*** (1.18)	4.70** (1.51)
Forgone preventive care	2.58*** (0.50)	3.41*** (0.59)
Forgone other types of care	0.52 (0.40)	0.41 (0.47)
Asian × forgone chronic care (ref = no foregone care)		1.10 (1.51)
Asian × forgone mental care		5.69 (5.44)
Asian × forgone preventive care		− 2.91 (2.07)
Asian × forgone other types of care		2.88^+^ (1.63)
Black × forgone chronic care (ref = no foregone care)		2.10^+^ (1.10)
Black × forgone mental care		− 0.55 (4.27)
Black × forgone preventive care		− 0.41 (1.67)
Black × forgone other types of care		0.62 (1.17)
Hispanic × forgone chronic care (ref = no foregone care)		− 1.63 (1.08)
Hispanic × forgone mental care		1.56 (2.60)
Hispanic × forgone preventive care		− 6.29*** (1.68)
Hispanic × forgone other types of care		− 1.35 (1.46)
Panel B: poor sleep quality		
Forgone chronic care (ref = no foregone care)	0.76*** (0.16)	0.51** (0.19)
Forgone mental care	1.69** (0.58)	1.69** (0.65)
Forgone preventive care	1.05*** (0.22)	1.18*** (0.25)
Forgone other types of care	0.39* (0.17)	0.49** (0.20)
Asian × forgone chronic care (ref = no foregone care)		− 0.93 (0.64)
Asian × forgone mental care		0.03 (2.32)
Asian × forgone preventive care		− 0.79 (0.89)
Asian × forgone other types of care		− 0.46 (0.69)
Black × forgone chronic care (ref = no foregone care)		1.41** (0.48)
Black × forgone mental care		− 0.60 (2.39)
Black × forgone preventive care		0.92 (0.75)
Black × forgone other types of care		0.44 (0.51)
Hispanic × forgone chronic care (ref = no foregone care)		− 0.64 (0.48)
Hispanic × forgone mental care		− 1.46 (1.74)
Hispanic × forgone preventive care		− 1.19 (0.72)
Hispanic × forgone other types of care		− 1.16^+^ (0.62)

Model 1 was the base model. Model 2 included interaction terms between missed care across various types and measures of psychological well-being. Coefficients for covariates were omitted for brevity

^*^p < 0.05; **p < 0.01; ***p < 0.001; ^+^*p* < 0.1

## Discussion and Implications

The COVID-19 pandemic has significantly altered the healthcare experiences of older adults, resulting in a significant number of instances of forgone care. However, there is still limited understanding of how these experiences have contributed to the burden and stress experienced by older adults, and how they have impacted their psychological well-being. Furthermore, the psychological impact on minoritized older adults who experienced forgone care has not been explored in-depth. To address this critical issue, this study utilizes nationally representative HEAP data, which includes detailed information on various dimensions of forgone care experiences among older adults and has oversampled racial and ethnic minorities.

This study has four key findings. First, forgone care experiences were prevalent across all racial and ethnic groups. Approximately two-thirds of older adults missed or delayed healthcare due to COVID-19, which is higher than what previous studies have found [25]. Second, having forgone care was significantly linked to higher psychological distress and poorer sleep quality among older adults, with some racial and ethnic variations. Hispanics exhibited less psychological distress from forgone preventive care compared to Whites, while Blacks experienced poorer sleep quality than their White counterparts in relation to a variety of forgone care experiences. Third, the accumulation of missed care types was positively correlated with psychological distress and poor sleep quality, with Blacks again showing a greater decline in sleep quality compared to Whites. Finally, in terms of specific types of forgone care, this study found that missed chronic care, mental care, and preventative care were all consequential for psychological well-being. Nevertheless, Hispanic older adults showed less distress from missed preventative care and Blacks experienced a greater decline in sleep quality from missed chronic care. Taken together, medical research has long acknowledged the interplay between physical and mental health. Forgone care may be a critical mechanism through which untreated or delayed physical health issues lead to an increased risk of mental health problems.

Several findings have enormous implications for geriatrics practices. First, this study finds that although the prevalence of forgone care was more or less similar across racial and ethnic groups, Black individuals were associated with worsened sleep quality. The finding is consistent with the literature that minority populations tend to be more affected by adverse social structural and healthcare contexts [26, 27] and disruptions due to the COVID-19 pandemic [28]. This may be because Black older adults who experienced forgone care are also likely to experience greater disruptions in other aspects of their lives, including food security and economic security [29], even though prior studies suggest that Black older adults hold more psychological resources and resilience [30, 31]. Furthermore, Black communities have higher rates of chronic conditions such as hypertension, diabetes, and obesity [32]. These conditions increase the risk of severe outcomes from COVID-19, including hospitalization and death, which may contribute to poorer sleep quality among Black older adults who experienced forgone care. Therefore, addressing the comprehensive needs of Black older adults during the pandemic is crucial to narrowing the gap in poor sleep quality.

Second, the similarity in the experience of forgone care across different racial and ethnic groups during the pandemic, as well as the no difference in the associations between forgone care and psychological well-being during the pandemic, warrants further discussion. These somewhat surprising findings first suggest that not all aspects of inequality, such as disparities in healthcare access, necessarily manifest more sharply during a crisis like the COVID-19 pandemic [33]. Our results imply that the pandemic created a level of disruption to healthcare systems—such as clinic closures, delays in elective procedures, and a general strain on healthcare resources—that potentially affected all older adults in a more uniform way, regardless of race or ethnicity. In addition, we found that most forgone care experiences were associated with mental health outcomes similarly across all racial and ethnic groups. It is possible that the pandemic brought unprecedented public attention to the concept of forgone care, making it a shared concern across society. As a result, individuals from all racial and ethnic groups may have experienced similar feelings of frustration, uncertainty, or helplessness tied to unmet healthcare needs, contributing to comparable mental health outcomes. Alternatively, differences in sensitivity to forgone care could result in balancing effects across groups, leading to similar psychological consequences. For example, White individuals, who may have higher expectations for consistent healthcare access, might experience greater distress when care is forgone. In contrast, Black individuals, who often face systemic barriers [34], might be less sensitive to these disruptions, as such experiences align with pre-existing inequities. These differing responses could balance out, resulting in broadly similar mental health consequences across groups.

Finally, much of the forgone care likely occurred during the early months of the pandemic. The associations observed between these forgone healthcare opportunities and psychological well-being—measured within a one-week or one-month timeframe—may reflect lasting, long-term effects on mental health. These enduring consequences could stem from unresolved health concerns or lingering uncertainty about one’s condition, which may continuously elevate stress levels and disrupt sleep quality. Another possibility is that symptoms worsened due to delayed or missed care earlier in the pandemic. Over time, untreated symptoms may accumulate. For example, missing scheduled medical appointments has been associated with poorer glycemic control in patients with diabetes [35]. Similarly, early-stage interventions for mental health conditions are critical for preventing more severe illness over time [36]. This can thus lead to more serious health conditions when older adults finally access care. These two mechanisms may help explain the observed relationship between earlier forgone care and more recently measured psychological well-being. Therefore, this study suggests that even as healthcare services resume, earlier experiences of forgone care may continue to significantly impact mental well-being, contributing to a more sustained and lingering psychological burden.

Before concluding, we acknowledge a few limitations. First, the HEAP study did not ask respondents about the frequency and reasons for forgone care. This prevented us from further analyzing the severity of forgone care experiences and whether the mental health consequences vary by reason for forgone care. Also, forgone care experiences may be underestimated due to the self-reported nature of the data. Additionally, because this is a cross-sectional study, we are not able to follow up on the long-term physical and mental health consequences of forgone care. There may be other potential unmeasured confounders, such as personal health behaviors, pre-existing mental health conditions, and socioeconomic status, which makes the results associational instead of causal. Finally, our findings only apply to the early phase of the pandemic and can only generalize to middle-aged and older adults in the United States. As the pandemic unfolds and government policies change, experiences of forgone care and its consequences may differ.

Overall, this study provides valuable insights into the patterns and consequences of forgone care among older adults during the COVID-19 pandemic. By understanding the determinants and impacts of forgone care, healthcare providers and policymakers can develop targeted strategies to address these barriers and ensure that vulnerable populations receive the care they need. Future research is necessary to assess the enduring impact of the pandemic on healthcare utilization and outcomes, with an emphasis on creating robust healthcare systems capable of withstanding future public health crises.

## Supplementary Information

Below is the link to the electronic supplementary material.

Supplementary file1 (DOCX 429 KB)
