# Supplementary figures and images for: Experiences of Forgone Care During the COVID-19 Pandemic and Older Adults’ Mental Health: Variations by Race and Ethnicity
Source: J Racial Ethn Health Disparities. 2025 Feb 11;13(2):929–36. doi: 10.1007/s40615-025-02304-0 (PMC12966222; doi:10.1007/s40615-025-02304-0)

Supplemental Figure 1


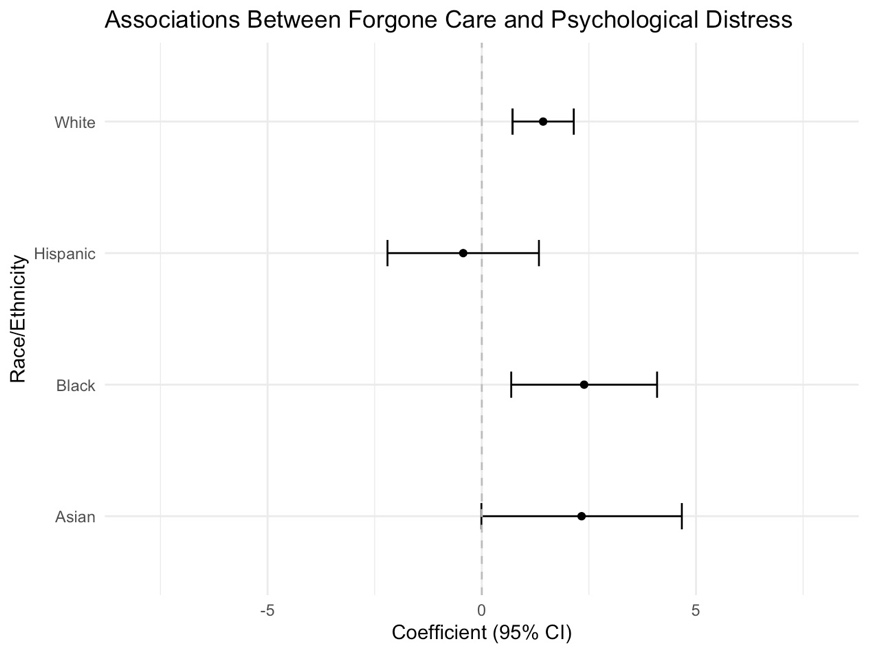

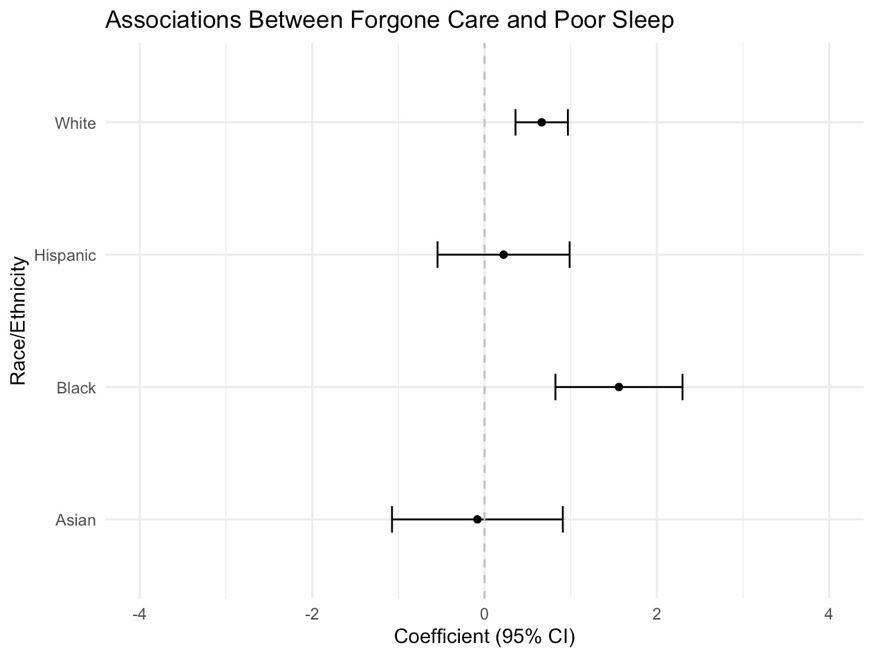


Supplemental Figure 2


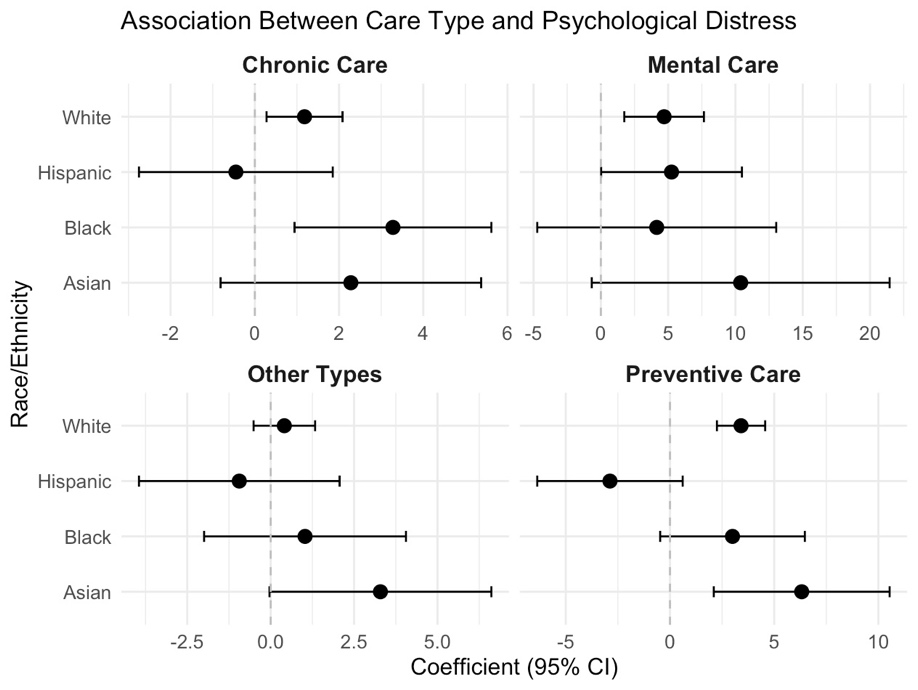

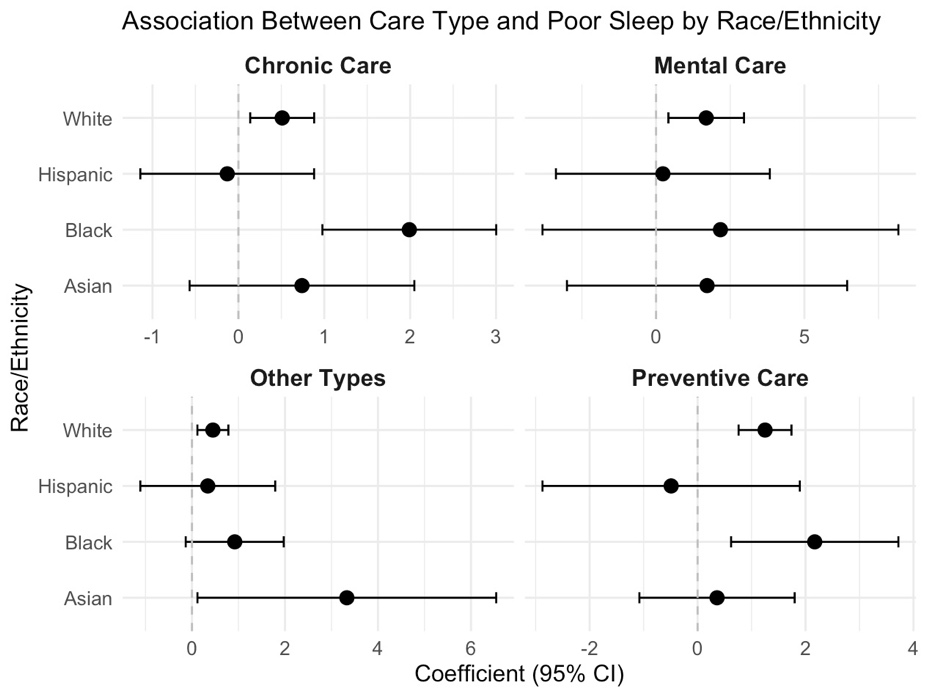


Supplemental Figure 3


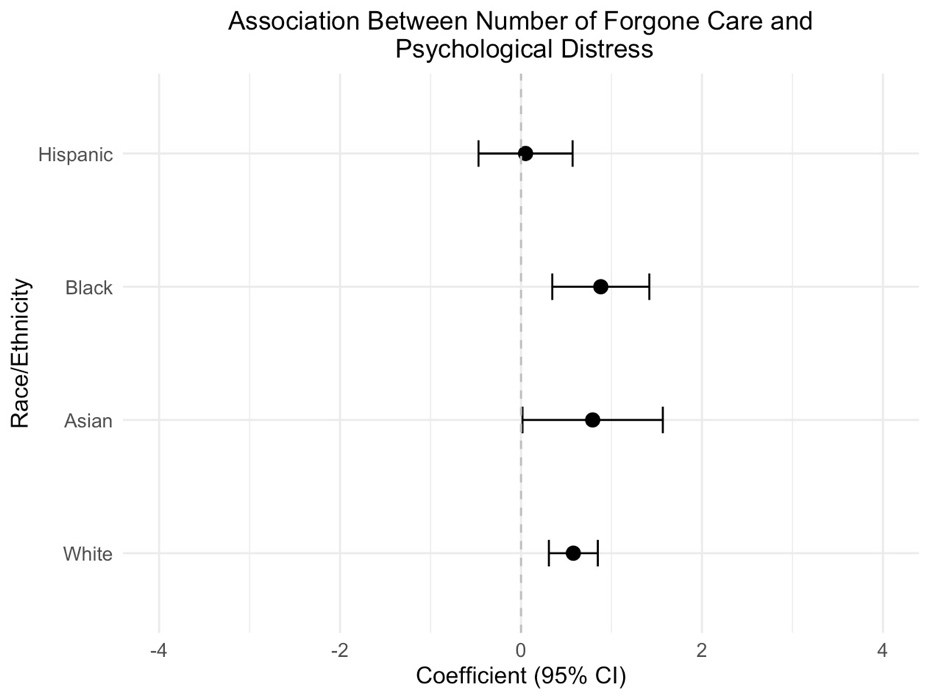

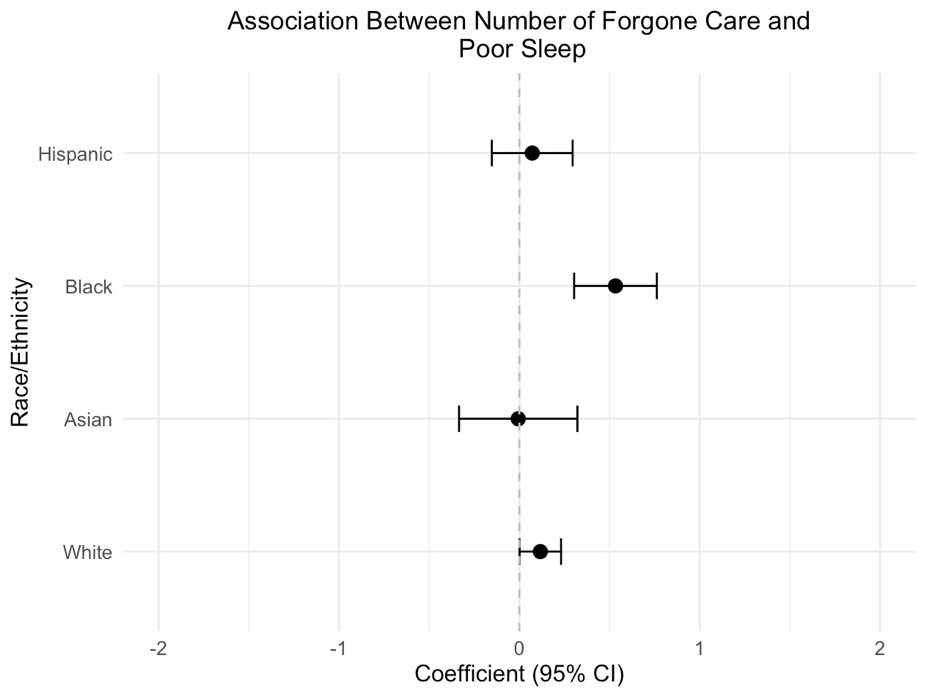

Supplement: Supplementary file 1 — Supplementary file1 (DOCX 429 KB) [file 40615_2025_2304_MOESM1_ESM.docx]
